# Fast Dynamic *in vivo* Monitoring of Erk Activity at Single Cell Resolution in DREKA Zebrafish

**DOI:** 10.3389/fcell.2018.00111

**Published:** 2018-09-25

**Authors:** Vanessa Mayr, Caterina Sturtzel, Manuela Stadler, Sarah Grissenberger, Martin Distel

**Affiliations:** Innovative Cancer Models, St. Anna Kinderkrebsforschung, Children's Cancer Research Institute, Vienna, Austria

**Keywords:** Zebrafish (*Danio rerio*), signaling pathway activation, ERK activity dynamics, *in vivo* imaging, *in vivo* pharmacology, wounding

## Abstract

Precise regulation of signaling pathways in single cells underlies tissue development, maintenance and repair in multicellular organisms, but our ability to monitor signaling dynamics in living vertebrates is currently limited. We implemented kinase translocation reporter (KTR) technology to create DREKA (“**d**ynamic **r**eporter of **E**r**k a**ctivity”) zebrafish, which allow one to observe Erk activity *in vivo* at single cell level with high temporal resolution. DREKA zebrafish faithfully reported Erk activity after muscle cell wounding and revealed the kinetics of small compound uptake. Our results promise that kinase translocation reporters can be adapted for further applications in developmental biology, disease modeling, and *in vivo* pharmacology in zebrafish.

## Introduction

Signaling pathways underlie cellular behavior during development, repair and disease, but fully understanding the function of any one pathway requires one to follow its dynamic activity within the context of single cells in tissues of a whole living organism. An essential pathway for cell proliferation and differentiation is the evolutionarily conserved mitogen activated protein kinase (MAPK) pathway, consisting of a three kinase phosphorylation relay cascade (e.g., RAF/MEK/ERK) (Krens et al., 2006). Dysregulation of the MAPK pathway can lead to severe developmental abnormalities and diseases (Kim and Choi, 2010; Rauen, 2013; Burotto et al., 2014). Hyperactivating mutations in the RAS/MAPK pathway underlie a group of developmental disorders like Costello or Noonan Syndrome, commonly termed RASopathies, and also occur in many types of cancer, including melanoma (BRAFV600E mutation) and colon cancer (K-RASG12D/G12V) (Forrester et al., 1987; Davies et al., 2002; Rauen, 2013). The RAS/RAF/MEK/ERK signaling cascade has therefore become a major drug target in various cancers and inhibitors for RAF, MEK and ERK are available (Girotti et al., 2015).

The need to understand MAPK signaling activation in normal and disease states has led to the development of live reporters for visualization of kinase activity. Sensors based on Förster resonance energy transfer (FRET) provide great insights by visualizing ERK activity in cultured cells and more recent also in mice and zebrafish, but are difficult to implement and fail to accurately report the downregulation of activity (Vandame et al., 2013; Regot et al., 2014; Depry et al., 2015; Hirata et al., 2015; Hiratsuka et al., 2015; Sari et al., 2018). Regot et al. recently introduced an alternative kinase activity reporter termed kinase translocation reporter (KTR) and demonstrated its high sensitivity *in vitro* (Regot et al., 2014). This technology translates a phosphorylation event into a nucleo-cytoplasmic shuttling event of the synthetic reporter, which can easily be observed by fluorescence microscopy. We reasoned that transferring KTR technology to zebrafish would result in novel vertebrate kinase activity reporters with unprecedented temporal resolution and sensitivity. Due to its transparency and external development, zebrafish is ideally suited for *in vivo* fluorescence microscopy investigations. Current zebrafish pathway reporters like the FGF reporter *Tg(Dusp6:d2EGFP)*^*pt*^^6^ strain are based on expression of destabilized fluorescent proteins with a half-life of ~2 h (Molina et al., 2007). Still, fast and dynamic changes in signaling activity cannot be visualized by such reporters. Here, we generated a KTR-based Erk activity reporter zebrafish strain (DREKA) and successfully demonstrated its ability to visualize fast Erk signaling dynamics in a wound response and its possible application for *in vivo* pharmacology.

## Materials and methods

### Maintenance of fish

Zebrafish (*Danio rerio*) were maintained at standard conditions (Kimmel et al., 1995; Westerfield, 2000) according to the guidelines of the local authorities under licenses GZ:565304/2014/6 and GZ:534619/2014/4.

### Plasmid construction

The DREKA transgenesis vector #260 (pDESTubi:ERK-KTR-CloverpATol2) was generated by Gateway® recombination using p5‘ubiquitin, pSR1835 containing (pENTR)ERK-KTR-Clover (addgene #59138), p3‘pA and pDestTol2pA4 vectors.

The T55L/T62L reporter was created using Gibson assembly (NEBuilder Hifi DNA assembly cloning kit, E5520, New England BioLabs GmbH, Frankfurt, Germany) of PCR fragments amplified from #260 using the following primer pairs:

639_ubiERK-TtoL1R: ACGTGGCTTCTTCGATGGcagCGCTG/

513: CATTTGGACAATTTTGCTGCAGGTAAAATGC and

640_ubiERK-TtoL2-F: tgCCATCGAAGAAGCCACGTctgCCATC/

516: TCGCCCTTGCTCACCATACTAGTGGA and ligated into #260 opened by PstI/SpeI digest (New England BioLabs GmbH, Frankfurt, Germany).

The T55V/T62V reporter was created using Gibson assembly (NEBuilder Hifi DNA assembly cloning kit, E5520) of PCR fragments amplified from #260 using the following primer pairs:

641_ubiERK-TtoV1-R: ACGTGGCTTCTTCGATGGcacCGCTG/

513: CATTTGGACAATTTTGCTGCAGGTAAAATGC and

642_ubiERK-TtoV2-F: tgCCATCGAAGAAGCCACGTgtgCCATC/

516: TCGCCCTTGCTCACCATACTAGTGGA and ligated into #260 opened by PstI/SpeI digest.

The T55D/T62D reporter was created using Gibson assembly (NEBuilder Hifi DNA assembly cloning kit, E5520) of PCR fragments amplified from #260 using the following primer pairs:

517:ubiERK-TtoD1-R: ACGTGGCTTCTTCGATGGGTCCGCTG/513: CATTTGGACAATTTTGCTGCAGGTAAAATGC and

518: ubiERK-TtoD2-F: acCCATCGAAGAAGCCACGTgacCCATC/

516: TCGCCCTTGCTCACCATACTAGTGGA and ligated into #260 opened by PstI/SpeI digest.

### *in vitro* transcription of RNA

RNA for microinjection was transcribed *in vitro* using the Invitrogen^TM^ mMessage mMachine^TM^ SP6 transcription kit according to the manufacturer's recommendations (Ambion, AM1340, Waltham, MA, USA).

### Microinjection for transient assays and generation of transgenic strains

DNA/RNA injection was performed using injection capillaries (glass capillaries GB100F-10, with filament, Science Products GmbH, Hofheim, Germany) pulled with a needle puller (P-97, Sutter Instruments, Novato, USA) mounted onto a micromanipulator (M3301R, World Precision Instruments Inc., Berlin, Germany) and connected to a microinjector (FemtoJet 4i, Eppendorf, Hamburg, Germany).

For transient assays, fertilized Sanger AB Tübingen (SAT) eggs were injected with 25 pg pDESTubi:ERK-KTR-CloverpATol2 and 20 pg H2B-CFP mRNA.

MAPK pathway activation experiments were carried out by co-injecting KalTA4 mRNA (20 pg), H2B-CFP:UAS:HRASG12V (20 pg), and pDESTubi:ERK-KTR-CloverpATol2 (25 pg) at the one cell stage.

To create transgenic zebrafish, 20 pg Tol2 mRNA and 25 pg pDESTubi:ERK-KTR-CloverpATol2 were injected into fertilized SAT eggs at the one cell stage.

mClover expressing embryos were raised to adulthood and screened for germline transmission.

### Chemical inhibition

Transiently ERK-KTR-Clover expressing SAT or DREKA zebrafish embryos were dechorionated and incubated in the following compounds from 29 to 48 hpf: PD98059 (30 μM), vemurafenib (10 μM), PD0325901 (5 μM), trametinib (10 μM), and ulixertinib (1 μM). Stock solutions of compounds were in DMSO and control experiments were carried out in 0.1% DMSO. All compounds were purchased via MedChemTronica, Stockholm, Sweden with the respective ordering numbers HY-12028, HY-12057, HY-10254, HY-10999, HY-15816. Images were recorded at 48 hpf and Erk activity status was analyzed (*n* = 5 embryos each condition, except for vemurafenib *n* = 4 embryos).

Leptomycin B (Cat No. L2913, Sigma Aldrich, Saint Louis, USA) treatment was carried out from 26 hpf for 24 h at 92 μM. Leptomycin B stock solution was in 70% methanol and control experiments were carried out in 0.7% methanol/E3.

### Imaging

Zebrafish embryos were prepared for imaging as described previously. (Distel and Köster, 2007). In brief, zebrafish embryos were dechorionated, anesthetized using 1x tricaine in E3 medium (0.16 g/l tricaine (Cat No. E1052110G, Sigma-Aldrich Chemie GmbH, Steinheim, Germany), adjusted to pH 7 with 1M Tris pH 9.5, in E3), and embedded in 1.2% ultra-low gelling agarose (Cat. No. A2576-25G, Sigma-Aldrich Chemie GmbH, Steinheim, Germany) in a glass bottom imaging dish (D35-14-1.5-NJ, Cellvis, Mountain View, CA, USA). Images and time-lapse movies were recorded on a Leica SP8 X WLL confocal microscope system.

### Image and movie rendering

Images were rendered using Photoshop CS6 (Adobe), Leica LAS X software, Quicktime Pro and Fiji.

### Needle induced wounding

Zebrafish embryos were anesthetized in 1x tricaine/E3, embedded in 1.2% ultra-low gelling agarose in an imaging dish and manually wounded by introducing a small puncture using an injection needle.

### Laser induced wounding

Zebrafish embryos were embedded for imaging as described above. A laser-inflicted wound was introduced using the Leica SP8 X FRAP module and laser lines 405, 458, 476, and 488 nm simultaneously at ~75% laser power. A region of interest was selected manually and was illuminated for 80 to 90 s. After ~40 s a wound started to appear.

### Compound kinetics experiments

To investigate compound uptake kinetics, zebrafish embryos (55 hpf) were embedded in 1.2% ultra-low gelling agarose containing 10 μM trametinib. Embedded embryos were covered with 10 μM trametinib in 1x tricaine /E3/PTU (Cat. No. P762925G, Sigma- Aldrich GmbH, Steinheim, Germany) and imaged continuously on a Leica SP8 X WLL system.

### Quantification of ERK signaling activity

To quantify Erk signaling activity, Clover intensity was measured in the nucleus and in the cytoplasm of cells by selecting the respective region using the intensity v time monitor tool of the time series analyzer plugin for Fiji (J. Balaji, UCLA). The cytoplasmic to nuclear intensity ratio was calculated using Microsoft Excel.

### Immunofluorescence

Wounded and control zebrafish embryos (48 hpf) were fixed in 4% paraformaldehyde/PBS (Cat. No 15710-S, Electron Microscopy Sciences, Hatfield, PA, USA) for 4 h at room-temperature. Afterwards zebrafish embryos were transferred into 100% methanol and were incubated at −20°C overnight. Then embryos were transferred into acetone (7 min at −20°C) and incubated in H_2_O (room-temperature for 1 h). After washing with PBST (PBS with 0.1%Tween20) embryos were incubated in 150 mM TrisHCl (pH 9) (70°C for 15 min). After washing in PBST embryos were blocked in 10% normal goat serum (NGS) in PBST. Samples were incubated in p-ERK antibody (Cell Signaling technology, Cat. No. #4370) at 1:400 in 10% NGS in PBST overnight at 4°C. The primary antibody was removed and embryos were washed in PBST (6 × 15 min). A secondary antibody, Alexa Fluor 568 goat anti-rabbit antibody (Cat. No. A-21069, Invitrogen) was used at 1:2,000 together with DAPI in 10% NGS in PBST for overnight incubation at 4°C. After this incubation step embryos were washed in PBST (6 x 15 min) and imaged.

## Results

**Figure 1:**
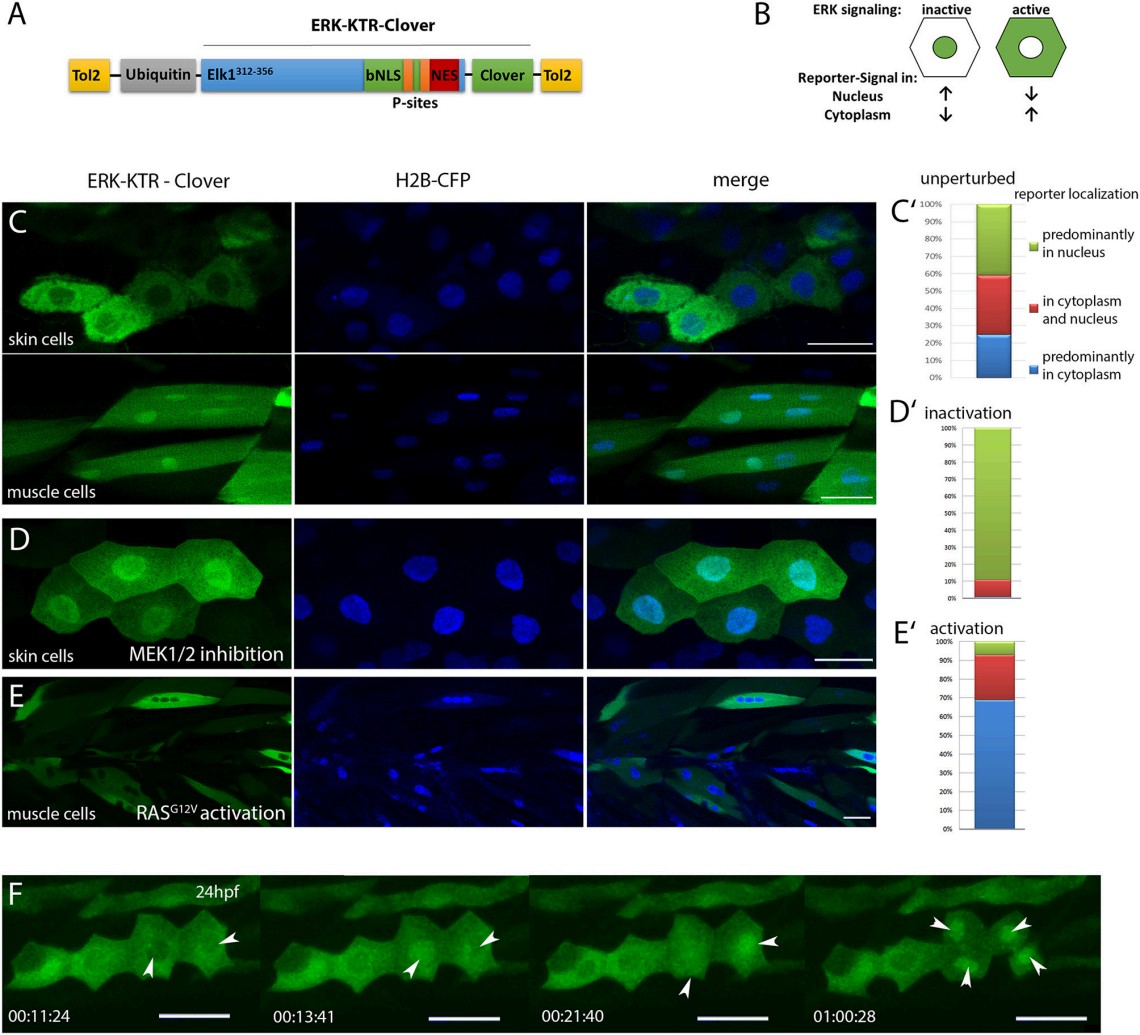
Characterization of ubi:ERK-KTR-Clover in zebrafish embryos. **(A)** Schematic illustration of the DREKA transgenesis vector pDESTubi:ERK-KTR-CloverpATol2. (Tol2, Tol2 recombinase recognition sites; ubiquitin, ubiquitin promoter; Elk1^312−356^, ERK docking sites from Elk1; bNLS, bipartite nuclear localization signal; P-sites, phosphorylation sites; NES, nuclear export signal; modified after Regot et al., 2014) **(B)** Schematic depiction of the ERK-KTR reporter principle. ERK signaling inactive: reporter is mainly localized in the nucleus; ERK signaling active: reporter is localized in the cytoplasm sparing the nucleus. **(C)** pDESTubi:ERK-KTR-CloverpATol2 and H2B-CFP mRNA co-injected wildtype zebrafish embryos express ERK-KTR-Clover in a mosaic manner. mClover fluorescence is mainly found in the cytoplasm of skin epithelial cells, indicating active Erk signaling (upper panel) and in the nucleus of muscle cells indicating absence of Erk signaling activity (lower panel). **(C**′**)** Quantification of ERK-KTR localization in skin epithelial cells and muscle cells at 48 hpf. Green, mClover fluorescence in the nucleus; Red, mClover fluorescence distributed throughout the cell; Blue, mClover fluorescence in the cytoplasm sparing the nucleus (*n* = 385 cells, 10 embryos at 48 hpf). **(D)** Skin epithelial cells of co-injected embryos incubated with MEK1/2 inhibitor PD98059 (30 μM) overnight show mClover fluorescence in the nucleus at 48 hpf indicating the reporter responds to Mek inhibition. **(D**′**)** Quantification of mClover localization shows reporter shuttling to the nucleus after MEK1/2 inhibition (*n* = 300 cells, 5 embryos at 48 hpf). **(E)** Muscle cells expressing constitutively active HRAS (zebrafish injected with pDESTubi:ERK-KTR-CloverpATol2, H2B-CFP:UAS:HRASG12V, and KalTA4 mRNA Distel et al., 2009) show active Erk signaling at 48 hpf. **(E**′**)** Quantification of mClover localization shows constitutively active HRAS induced reporter shuttling to the cytoplasm (*n* = 235 cells, 5 embryos at 48 hpf). **(F)** Mitotic skin epithelial cells of pDESTubi:ERK-KTR-CloverpATol2 injected zebrafish at 24 hpf. Dividing skin epithelial cells (white arrowheads) show dynamic Erk signaling with a sudden change from cytoplasmic to nuclear reporter localization before cytokinesis [compare time points 11:24 min/13:41 min (left arrowhead) and 13:41 min/21:40 min (right arrowhead)]. After cytokinesis the reporter remains in the nuclei of both daughter cells (arrowheads at 1:00:28 h). Images taken from a time-lapse movie (Movie 2) are maximum projections of several planes. All scale bars are 25 μm. All images were recorded on a Leica SP8 X WLL confocal microscope and rendered using Photoshop CS6.

In order to generate a highly dynamic reporter for Erk activity, we aimed to adapt KTR technology for its application in zebrafish. The ERK reporter ERK-KTR-Clover consists of an ERK docking site fused to nuclear localization (NLS) and nuclear export signals (NES) and the fluorescent protein mClover. Upon phosphorylation of phospho-acceptor sites within the NLS the export signal overrides the import signal and the green fluorescent reporter localizes from the nucleus to the cytoplasm, hereby visualizing ERK activity (see Figure 1B; Regot et al., 2014).

We first investigated *in silico*, if zebrafish Erk1/2 would be able to bind to the reporter construct, which carries the minimal ERK specific docking site (F-site) of mouse Elk1 (FQFP), which is also present in *Danio rerio* Elk1 (Jacobs et al., 1999). ERK1/2 is generally well conserved between human, mouse and zebrafish and the F-site recruitment site (FRS), which binds to the F-site, is present in zebrafish, suggesting that the synthetic KTR construct will likely be able to report ERK activity in zebrafish (Figure S1) (Roskoski, 2012; Busca et al., 2016). To test this, we next placed ERK-KTR-Clover under control of the zebrafish *ubiquitin* promoter and transiently expressed the reporter in zebrafish embryos (Figure 1A; Mosimann et al., 2011; Regot et al., 2014). In cells of these embryos, ERK-KTR-Clover was found either in the cytoplasm sparing the nucleus, in the cytoplasm and nucleus or predominantly in the nucleus as confirmed by co-expression with the nuclear marker histone2B–CFP (H2B-CFP), indicating various degrees of Erk activity (Distel et al., 2010). We also observed cell type specific differences in reporter localization, e.g., skin epithelial cells showed dynamic and generally higher reporter intensity in the cytoplasm, whereas muscle cells showed stronger reporter signal in the nucleus at 48 hpf (*n* = 385 cells, 10 embryos) (Figures 1C,C′). Control reporter constructs, where threonines within the NLS were replaced by either leucine (T55L/T62L) or valine (T55V/T62V) were found in nuclei of all cell types investigated and a phosphomimetic reporter variant T55D/T62D localized to the cytoplasm (*n* = 132 muscle cells, 3 embryos), indicating that the localization of the reporter is indeed regulated by phosphorylation in zebrafish (Figure S2).

We next tested if the ERK-KTR reporter responds to inactivation and activation of the MAPK pathway in skin epithelial and muscle cells. Applying a MEK inhibitor (30 μM PD98059) for 17 h resulted in nuclear localization of the reporter in the majority of cells at 48 hpf (*n* = 300 cells, 5 embryos) (Figures 1D,D′). In contrast, stimulation of Erk signaling by co-expression of a constitutively active RAS (H-RAS^G12V^) shifted reporter localization to the cytoplasm of muscle cells at 48 hpf (*n* = 235 cells, 5 embryos) (Figures 1E,E′). These results suggested that ERK-KTR-Clover faithfully reports Erk activity in skin epithelial and muscle cells in living zebrafish embryos.

Finally, we probed the temporal resolution of the reporter by recording time-lapse movies of ERK-KTR-Clover injected embryos. This revealed changes in Erk activity within minutes in skin epithelial cells over time (Movie 1). Interestingly, dividing cells showed a stereotypical pattern of the reporter shuttling to the nucleus right before cytokinesis and staying in the nuclei of both daughter cells afterwards, indicating low Erk activity after cell division (Figure 1F and Movie 2).

With these transient assays being successful, we next generated transgenic zebrafish *[Tg(ubi:ERK-KTR-Clover)*^*vi*1^*]*, expressing ERK-KTR-Clover under control of the rather weak, but ubiquitous *ubiquitin* promoter in order to be able to investigate Erk signaling over longer periods of time in a non-mosaic manner (Mosimann et al., 2011). We named *Tg(ubi:ERK-KTR-Clover)*^*vi*1^ “DREKA” for “**d**ynamic **r**eporter of **E**r**k a**ctivity.” DREKA zebrafish were viable, showed no obvious morphological defects and were fertile (now in F4), indicating that the reporter does not negatively affect endogenous Erk signaling. F2 DREKA were confirmed to report changes in Erk activity by applying different inhibitors of the MAPK pathway. As expected MEK inhibitors trametinib (10 μM) or PD0325901 (5 μM) and ERK inhibitor ulixertinib (1 μM) decreased Erk signaling activity in skin epithelial cells of DREKA embryos, but vemurafenib (10 μM) a type I BRAF inhibitor specific for mutant BRAF (BRAF^V600E^) did not (Figure S3). These results confirmed that DREKA report changes in Erk activity upon external manipulation of the MAPK pathway.

In cells of DREKA zebrafish, changes in reporter localization lead to changes in mClover fluorescence intensity in the cytoplasm (C) and the nucleus (N). Calculating the C/N intensity ratio is thus a way to visualize and quantify relative changes in Erk activity over time on the single cell level. In muscle and skin epithelial cells, the C/N ratio ranged approximately from 0.6 to 1.5 in untreated DREKA zebrafish. Shuttling of the reporter to the cytoplasm by nuclear export is believed to be Exportin dependent. Indeed, inhibiting Exportin 1 by leptomycin B treatment (92 μM for 24 h) led to accumulation of the reporter in the nucleus reaching C/N intensity ratios of up to 0.23–0.27 in muscle and skin cells (typically 0.6–0.7 in untreated zebrafish) (Figure S4; Kudo et al., 1999). This shows that the reporter concentration is not reaching saturation in the nucleus in these cell types in untreated DREKA fish.

We next aimed to apply DREKA to assess the temporal dynamics of Erk signaling in a biological process under experimental conditions with control over an external stimulus eliciting Erk signaling and turned to a wounding assay.

Upregulation and correct temporal orchestration of ERK activity is necessary for proper wound healing across species. In *Xenopus* embryos, two temporally distinct phases of wound healing have been observed: an early and fast phase with high Erk activity and a second slow phase with high PI3K activity (Li et al., 2013).

**Figure 2:**
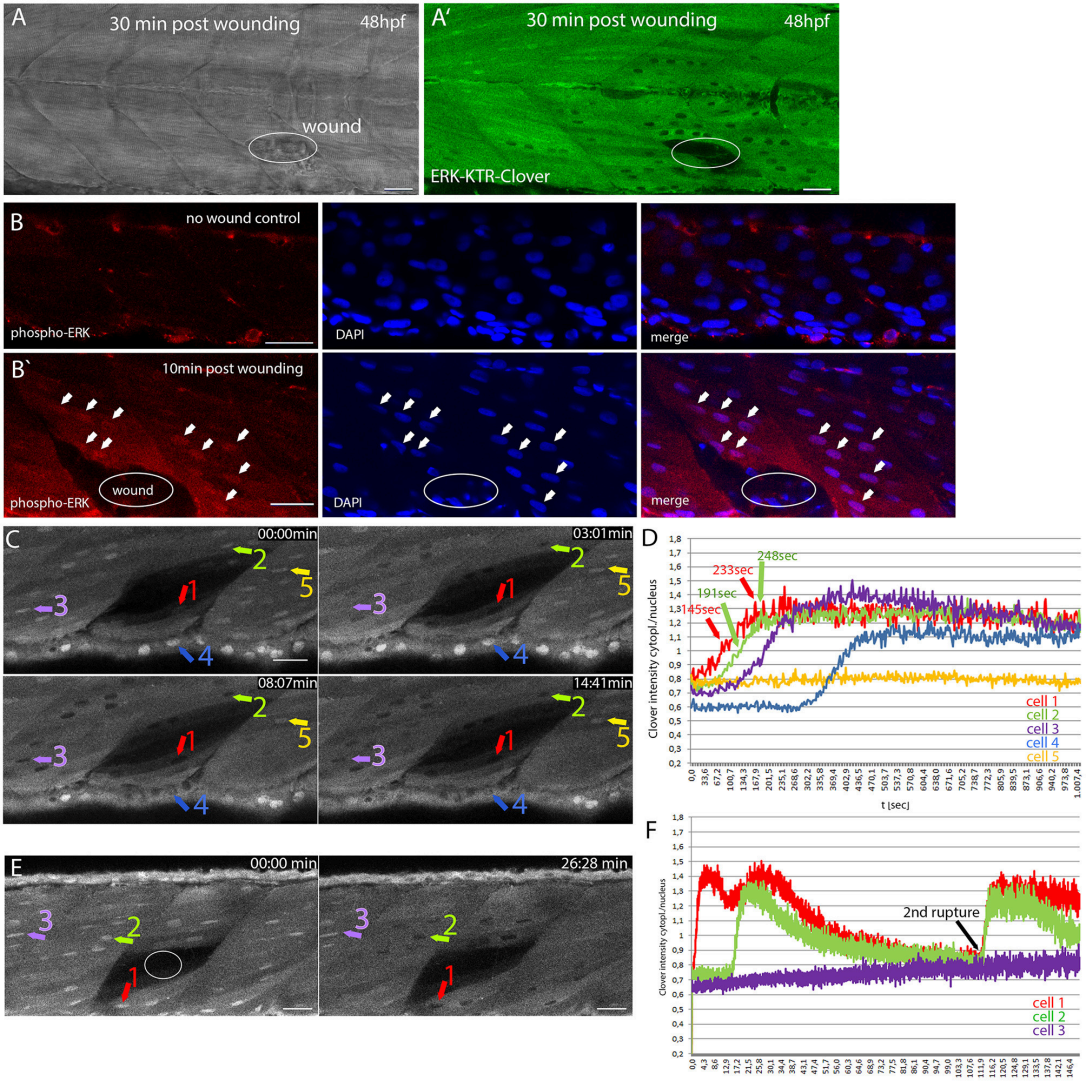
Monitoring dynamic Erk activity in DREKA zebrafish **(A)** Brightfield image and fluorescence image **(A**′**)** of a DREKA embryo wounded with a glass needle at 48 hpf. Images were taken 30 min after wounding. Active Erk signaling was observed in muscle cells around the wound (*n* = 6 embryos). **(B)** Immunofluorescence staining for phosphorylated Erk (red) in muscle cells of control and zebrafish embryos wounded with a glass needle 10 min post-wounding **(B**′**)**. DAPI staining is shown in blue. Arrows mark some of the cells with phosphorylated Erk being visible in the nucleus surrounding the wound (circle). **(C)** Erk signaling activation in muscle and skin epithelial cells after laser induced wounding in 54 hpf DREKA. Four time points (0, 3, 8, and 14 min) taken from time-lapse Movie 3 show the fast activation of Erk signaling. **(D)** Quantification of Erk activity in 5 cells as depicted in **(C)** starting ~65 s post-wound appearance. Erk activity is shown as cytoplasmic/nuclear ratio of mClover intensity over time (seconds). Cells close to the wound [#1(red) and #2(green)] show a fast response with higher reporter concentrations in the cytoplasm compared to the nucleus around 145 s (cell #1) and 191 s (cell #2) post-wound appearance and peaking after ~233 s (cell #1) and 248 s (cell #2), respectively. Cells further away respond later [#3(purple)] or remain inactive [#5(yellow)]. In addition to muscle cells, Erk signaling activation was also observed in skin epithelial cells [#4(blue)]. **(E)** Erk signaling activity in response to laser induced wounding of muscle cells in 72 hpf DREKA. mClover fluorescence (shown in gray) at 0 and 26 min after the wound (circle) has been introduced (see Movie 4). After wounding mClover starts to localize from the nucleus to the cytoplasm in cells directly adjacent to the wound, indicating Erk signaling activation (cell #1). Muscle cells further away from the wound become active for Erk signaling at later time points (cell #2) or remain inactive (cell #3). **(F)** Quantification of Erk activity in 3 cells as shown in **(E)** over 2.5 h starting ~40 s post-wound appearance (see Movie 4). Erk signaling activity is shown as cytoplasmic to nuclear mClover intensity over time (minutes) for one muscle cell close to the wound (#1 red), one muscle cell further away (#2 green), and one non-responding muscle cell (3# purple) (colored arrows in **E)**. Note the second rupture event leading to a rapid activation of Erk signaling. All scale bars are 25 μm. All images were recorded on a Leica SP8 X WLL confocal microscope and rendered using Photoshop CS6.

We asked if Erk signaling dynamics are similar in zebrafish embryos after wounding muscle cells. To verify that wounding activates Erk signaling, we punctured muscle tissue of 48 hpf DREKA embryos using a glass needle. Indeed, muscle cells close to the wound showed Erk signaling activation whereas muscle cells further away remained unaltered (*n* = 6 embryos; Figures 2A,A′). Erk signaling activity in muscle cells surrounding the wound was independently confirmed by immunofluorescence for phosphorylated Erk (*n* = 10 embryos; Figures 2B,B′). We next switched to a laser-induced wounding assay, which enabled us to follow Erk signaling changes continuously from the moment the wound was introduced. We detected an almost immediate response in neighboring cells after wounding of zebrafish muscle by high power laser illumination for 80–90 s (Figures 2C,D). The intensity of mClover in the cytoplasm increased steadily with the cytoplasmic/nuclear intensity ratio becoming >1 between 2 and 3 min and peaking around 4 min after wounding in fast responding cells (*n* = 7 cells, 3 embryos) (Figures 2C,D and Movie 3). Directly neighboring cells (Figure 2C, red arrow) responded first and subsequently Erk signaling activity also increased in cells further away (Figure 2C, purple arrow) from the wound. Spreading of Erk activity within one cell type (epithelial cells) was reported previously in mouse (Hiratsuka et al., 2015). Here, we observed two cell types, muscle cells and skin epithelial cells, relaying Erk signaling (Figures 2C,E and Movie 3, 4). After ~45 min muscle cells more distant to the wound started to become inactive for Erk signaling again and cells adjacent to the wound followed around 1 h after wounding (*n* = 6 embryos; Figures 2E,F). Intriguingly, we observed wounds, which spontaneously ruptured a second time after Erk signaling had already decreased, inducing another rapid, and simultaneous activation of Erk signaling in surrounding cells (Figure 2F and Movie 4). In such cases, live monitoring of Erk activity is of tremendous advantage as second rupture events would have likely been missed with current methods (e.g., immunofluorescence or Western Blotting), complicating the interpretation of the Erk signaling pattern (Movie 4). Wounding muscle cells in the presence of MEK1/2 inhibitors trametinib (10 μM, Movie 5) or TAK-733 (10 μM, Movie 6) did not elicit a translocation of the reporter as observable in 0.1% DMSO control experiments (Movie 7) (*n* = 4 embryos each), confirming that changes in reporter localization are MAPK dependent in our muscle wounding assay.

**Figure 3:**
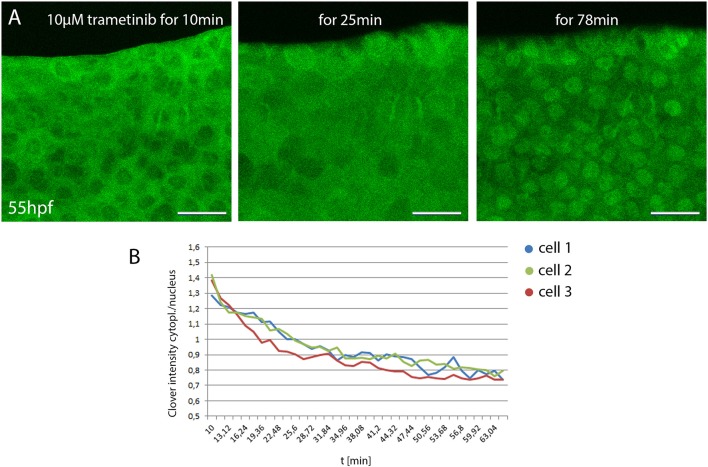
Monitoring small compound uptake kinetics in DREKA zebrafish **(A)** 55 hpf DREKA embryos were embedded in agarose containing 10 μM trametinib to investigate compound uptake kinetics in skin epithelial cells. After 10 min skin epithelial cells still show active Erk signaling. Around 20 min Erk activity is visibly decreased and by 50–60 min cells show full Erk signaling inhibition. **(B)** Quantification of Erk activity over time. Cytoplasmic to nuclear mClover intensity over time in three representative skin epithelial cells in DREKA zebrafish treated with 10 μM trametinib at 55 hpf. All scale bars are 25 μm. All images were recorded on a Leica SP8 X WLL confocal microscope and rendered using Photoshop CS6.

We next assessed the use of DREKA for *in vivo* pharmacology. Zebrafish is a popular model organism for small compound screening due to the ease of compound administration to the water. However, the kinetics of compound uptake, although of great importance are often unknown. To reveal uptake kinetics DREKA fish were exposed to 10 μM trametinib (MEK 1/2 inhibitor) and Erk activity was continuously monitored in skin epithelial cells, which showed predominantly active Erk signaling in their unperturbed state (Figure 3A). Ten minutes after treatment Erk signaling activity was still strong, however already after ~20 min a significant reduction in activity was detected with full inhibition being visible after around 50–60 min (*n* = 6 embryos) (see Figure 3 and Movie 8). Only mitotic skin cells maintained active Erk signaling, a finding that is consistent with previous research describing the insensitivity of mitotic cells to Mek inhibition (Hiratsuka et al., 2015). This indicates the potential of DREKA zebrafish and KTR technology for *in vivo* pharmacology at cellular resolution.

## Discussion

Recently, KTRs were introduced to report kinase activity with higher sensitivity and higher temporal resolution than commonly used kinase fusion or FRET based reporters *in vitro* (Regot et al., 2014). KTRs were also successfully applied in *C. elegans* (de La Cova et al., 2017). Here, we demonstrate that KTR technology can be transferred to a vertebrate model organism for real-time kinase activity monitoring. We created the zebrafish Erk activity reporter *tg(ubi:ERK-KTR-Clover)*^*vi*28^ (DREKA), containing an ERK-KTR, reported to not cross-react with p38 or JNK (Regot et al., 2014). DREKA zebrafish are viable, show no morphological abnormalities and are fertile (now in F4), indicating that ERK-KTR does not have adverse effects on zebrafish development when expressed under the *ubiquitin* promoter. We show that ERK-KTR faithfully reports changes in Erk activity in zebrafish skin epithelial cells and muscle cells by chemical and genetic perturbation as well as using phosphomimetic and “phospho-dead” reporter constructs. The dynamic range of this KTR appears well suited for measurements in muscle and skin cells with a relatively large cytoplasm. The cytoplasmic to nuclear (C/N) reporter intensity ratio, used as readout for Erk activity, typically ranges from 0.6 to 1.5, allowing one to monitor a broad range of Erk activity without saturation in skin and muscle cells. Whether this holds true for all cell types in the developing zebrafish needs further investigation as observed minimal and maximal C/N intensities vary between different cell types. In fact, in contrast to *C. elegans*, in zebrafish neural cells within the CNS, the reporter localized predominantly to the cytoplasm at all investigated time points. However, (T55L/T62L) and (T55V/T62V) reporter variants were present in nuclei of neural cells within the CNS (Figure S2). This suggests that either due to its high sensitivity ERK-KTR is already reporting very weak Erk activity in neural cells or that the C/N baseline ratio of reporter localization is shifted in this cell type. Multiple factors including cellular concentrations of nuclear import/export machinery proteins (e.g., importins, exportins, and Ran proteins) as well as phosphatases and cell morphology (cytoplasmic to nuclear volume ratio) can influence the localization and the relative intensity of the reporter in the respective compartment and thus the C/N ratio. Therefore, optimizing the NLS for reporter usage in neural cells in zebrafish, similar to modification attempts for *C. elegans* could improve the ERK activity reporter for this cell type (de La Cova et al., 2017). In addition, second generation constructs including a nuclear marker being co-expressed with the KTR can be used to solely measure changes in nuclear reporter intensity (de La Cova et al., 2017).

Although no absolute values of Erk activity can currently be stated, the generated transgenic Erk reporter strain (DREKA) offers unprecedented temporal resolution for monitoring changes in Erk activity in specific zebrafish cell types. State-of-the-art reporter strains are based on expression of destabilized fluorescent proteins. Thus, they suffer from delays reporting the onset and offset of signaling activity and are not capable of reporting fast dynamic signaling processes. In contrast, a readout based on nucleo-cytoplasmic shuttling of the fluorescent KTR reporter is not subject to limitations imposed by protein expression and stability rates.

In our wounding assay we observed an immediate increase (within seconds) of the cytoplasmic to nuclear reporter distribution, indicating Erk signaling activation with a peak after ~4 min. Previously, it had been reported that the Ras/Erk signaling module acts as a high-bandwidth and low-pass filter with the need for an external stimulus to persist for at least 4 min to activate the Ras/Erk module *in vitro* (Toettcher et al., 2013). If the faster response in our assay is due to actual differences between the *in vitro* and *in vivo* situation or if activation dynamics are cell type or stimuli specific, needs further investigations. Sensitivity differences of the used ERK reporter might also play a role. Consistent with possibly faster responses, Erk activity was found to be active 2 min after wounding in a *Xenopus* embryo wound assay based on Western blot analysis of Erk phosphorylation (Li et al., 2013). In *Xenopus*, Erk remained active between 30 and 60 min matching our observation in DREKA fish. This suggests that Erk activity dynamics might be similar in response to wounding across different cell types and developmental time points in *Xenopus* and zebrafish.

As pilot experiments indicated the feasibility of differential Erk activity analysis in selected cells during zebrafish development, we envision that DREKA fish will be useful for developmental biologists to decipher when and where Erk activity is needed for proper tissue formation. Here, we have also generated an UAS:ERK-KTR variant to be readily combined with available Gal4 zebrafish strains for tissue specific analysis of Erk activity. Moreover, KTR-enabled monitoring of Erk signaling activity in various mutant zebrafish and disease models, including cancer models, will reveal potentially altered signaling dynamics. Here, the development of software tools for automated analysis will soon be required due to the large datasets created by such experiments.

DREKA zebrafish and KTR technology also promise to be useful for pharmacological applications. For example effects of single or combination of compounds targeting Erk signaling can be investigated as we have shown by applying MEK, ERK, or RAF inhibitors (see Figure 3 and Figure S3). Off target effects of compounds, which are not primarily directed at the MAPK pathway will also be revealed. Although zebrafish is widely used in drug screening, the understanding of pharmacokinetics in zebrafish is currently lacking behind. A recent study applied liquid chromatography-mass spectrometry (LC-MS) based methods to determine paracetamol concentrations in zebrafish larvae (Kantae et al., 2016). Complementing such rather laborious approaches, DREKA offer a direct means to determine the time small compounds added to the water need to accumulate inside a cell at a concentration able to inhibit Erk activity as we demonstrated for trametinib (Figure 3). The ability to measure an effect on single cells within an intact organism in real time will also be beneficial to understand how drugs work *in vivo* and why they might fail. In comparison to conventional pharmacology approaches at tissue or organ level, single cell *in vivo* pharmacology is likely to enhance drug development (Vinegoni et al., 2015).

Finally, kinase translocation technology can be used to create reporters for various kinases, including JNK, p38, PKA, or AKT (Regot et al., 2014; Maryu et al., 2016). Multiplexing possibilities arise, which will allow one to dissect the interplay of various signaling pathways in a cell type specific way *in vivo*. This capability will likely have impact on our understanding of vertebrate development and disease.

## Ethics statement

All procedures involving animals were carried out according to EU guidelines and Viennese legislation (licenses: GZ:565304/2014/6 and GZ:534619/2014/4).

## Author contributions

VM and CS performed experiments, analyzed data, and wrote the manuscript. MS performed experiments. SG generated reporter constructs. MD designed and performed experiments, analyzed data, and wrote the manuscript.

### Conflict of interest statement

The authors declare that the research was conducted in the absence of any commercial or financial relationships that could be construed as a potential conflict of interest.

## Supplementary material

Figure S1 *In silico* cross-species analysis of Erk binding to ERK-KTR. **(A)** Alignment of zebrafish (*Danio rerio*) and mouse (*Mus musculus*) Elk1. The conserved FQFP motif of the F-site (ERK docking site) is shown in red. **(B)** Alignment of human, mouse and zebrafish Erk2. Amino acids M199, L200, Y233, L234, L237, and Y263 (human numbering), which form the F-site recruitment site are shown in red (Roskoski, 2012). Alignments were performed on ensembl.org.

Figure S2 Localization of ERK-KTR control constructs T55V/T62V and T55D/T62D. 48 hpf zebrafish embryos expressing either ERK-KTR-mClover or T55V/T62V and T55D/T62D control reporter constructs. **(A)** Neural cells within the CNS expressing ERK-KTR-mClover. The reporter localizes to the cytoplasm. **(B)** The T55V/T62V control reporter is also found in the nucleus of neural cells within the CNS. **(C)** Muscle cells expressing ERK-KTR-mClover. **(D)** The phosphomimetic T55D/T62D reporter construct localizes predominantly to the cytoplasm when expressed in muscle cells. All scale bars are 25 μm. Images were recorded on a Leica Sp8 X WLL confocal system and rendered with Adobe Photoshop CS6.

Figure S3 Effects of different inhibitors on Erk activity in DREKA zebrafish. Compounds were applied to F2 DREKA embryos at 29 hpf and quantification was performed on skin epithelial cells at 48 hpf. Percentage of cells with active Erk signaling shown as box plot from left to right: untreated control; ERK1/2 inhibitor: ulixertinib (1 μM); MEK1/2 inhibitor: trametinib (10 μM), PD0325901 (5 μM); B-RAF inhibitor: vemurafenib (10 μM); [each dot represents one embryo; box plot was created using R (r-project.org)].

Figure S4 Effects of nuclear export inhibition by leptomycin B. 26 hpf DREKA zebrafish larvae were treated with the nuclear export inhibiting compound leptomycin B at 92 μM for 24 h. **(A,C,E)** Treated DREKA, **(B,D,F)** untreated control DREKA. Muscle cells **(A,B)**, neural cells in the hindbrain **(C,D)**, and cranial ganglion cells **(E,F)** all show increased reporter signal in the nucleus in treated embryos compared to untreated siblings. Scale bars in **(A,B,D)** are 50 μm, in **(C,E,F)** 25 μm. Images were recorded on a Leica Sp8 X WLL confocal system using a 25x objective. Images were rendered using Adobe Photoshop CS6.

Movie 1 DREKA reveal dynamic Erk signaling in skin epithelial cells during development. Time-lapse movie of transiently ERK-KTR-Clover (green) expressing zebrafish embryos from ~48 to 54 hpf. Dynamic Erk activity can be observed as nuclear cytoplasmic shuttling of mClover in skin epithelial cells (see arrow). Z-stack images were recorded approximately every 3.5 min on a Leica SP8 X WLL confocal microscope using a 40x objective. Z-stacks are shown as maximum projections.

Movie 2 Erk activity in dividing cells. Time-lapse movie of transiently ERK-KTR-Clover (green) expressing zebrafish embryos from ~24 to 32 hpf. Dividing cells (arrows) show a stereotypical pattern of Erk signaling activity. Z-stack images were recorded approximately every 1 min on a Leica SP8 X WLL confocal microscope using a 40x objective. Z-stacks are shown as maximum projections.

Movie 3 Fast Erk signaling response in muscle and skin epithelial cells surrounding a wound. 54 hpf DREKA embryos were wounded by laser illumination. Erk signaling becomes active in muscle (white arrows) and skin epithelial cells (yellow arrow) surrounding the wound (circle). Single plane images were recorded starting ~65 s after wound appearance every 2.58 s on a Leica SP8 X WLL confocal microscope using a 40x objective.

Movie 4 Erk signaling in cells surrounding a wound. 72 hpf DREKA embryos were wounded by laser illumination (white circle). mClover fluorescence is shown in gray scale. Erk activity was monitored for 2.5 h after wounding. Spreading of Erk activity from the wound to muscle cells further away is visible. Note that both, muscle cells (red and green arrows) and skin cells (yellow arrow) react to wounding. After ~30–40 min Erk signaling activity decreases. Around 1 h 54 min the wound breaks open again activating Erk signaling in many cells simultaneously. Images were recorded starting ~40 s after wound appearance every 2.58 s on a Leica SP8 X WLL confocal microscope using a 40x objective.

Movie 5 Wounding response in presence of 10 μM trametinib. 26 hpf DREKA embryos were treated with 10 μM MEK1/2 inhibitor trametinib for 3 h and then embedded into ultra-low gelling agarose containing 10 μM trametinib. Larvae were locally wounded by laser illumination and mClover fluorescence in muscle cells was recorded. Erk activity was monitored in a time-lapse movie for at least 15 min after wounding. No Erk activation can be observed. Images were recorded on a Leica SP8 X WLL confocal microscope using a 40x objective.

Movie 6 Wounding response in presence of 10 μM TAK-733. 26 hpf DREKA embryos were treated with 10 μM TAK-733 for 3 h and then embedded into ultra-low gelling agarose containing 10 μM TAK-733. Larvae were locally wounded by laser illumination and mClover fluorescence in muscle cells was recorded. Erk activity was monitored in a time-lapse movie for at least 15 min after wounding. No Erk activation can be observed. Images were recorded on a Leica SP8 X WLL confocal microscope using a 40x objective.

Movie 7 Wounding response in presence of DMSO. As control to Movies 5, 6 26 hpf DREKA embryos were treated with 0.1% DMSO for 3 h and then embedded into ultra-low gelling agarose containing 0,1% DMSO. Larvae were locally wounded by laser illumination and mClover fluorescence in muscle cells was recorded. Erk activity was monitored for 20 min after wounding. After 90 s Erk activity is visible and spreading to neighboring muscle cells. Images were recorded on a Leica SP8 X WLL confocal microscope using a 40x objective.

Movie 8 Small compound uptake kinetics revealed by DREKA. 55 hpf DREKA embryos were embedded in agarose containing 10 μM trametinib to investigate compound uptake kinetics in skin epithelial cells. Approximately 10 min later a time-lapse movie of Erk activity was recorded. Initially, skin epithelial cells are still active. Around 25 min after compound administration Erk activity is visibly decreased and by 50 min cells show Erk signaling inhibition. Z-stack images were recorded approximately every 2 min for 1 h on a Leica SP8 X WLL confocal microscope using a 40x objective. Z-stacks are shown as maximum projections.
