# Supplementary figures and images for: Fast Dynamic in vivo Monitoring of Erk Activity at Single Cell Resolution in DREKA Zebrafish
Source: Front Cell Dev Biol. 2018 Sep 25;6:111. doi: 10.3389/fcell.2018.00111 (PMC6170801; doi:10.3389/fcell.2018.00111)

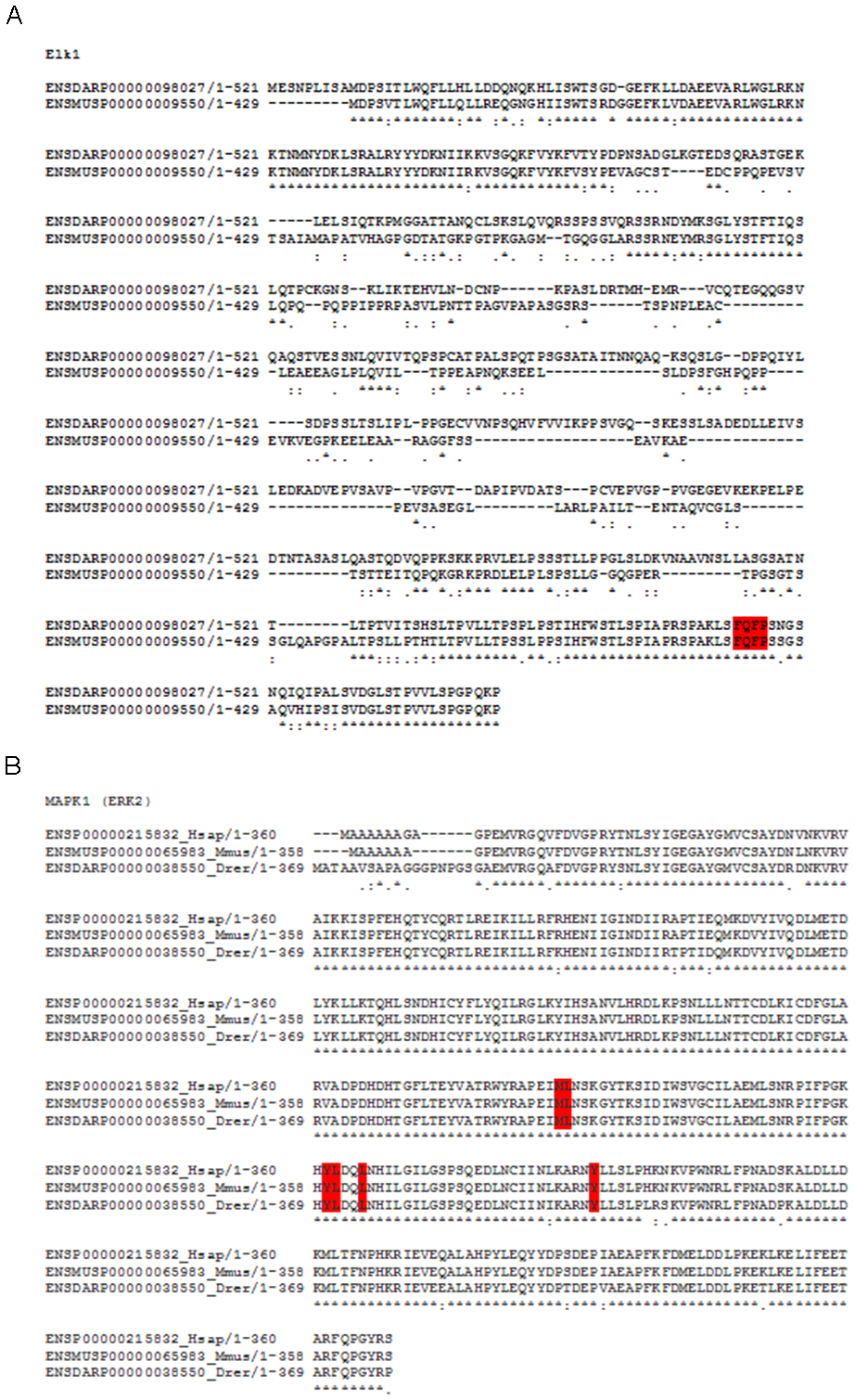

Supplement: Figure S1 — In silico cross-species analysis of Erk binding to ERK-KTR. (A) Alignment of zebrafish (Danio rerio) and mouse (Mus musculus) Elk1. The conserved FQFP motif of the F-site (ERK docking site) is shown in red. (B) Alignment of human, mouse and zebrafish Erk2. Amino acids M199, L200, Y233, L234, L237, and Y263 (human numbering), which form the F-site recruitment site are shown in red (Roskoski, 2012). Alignments were performed on ensembl.org. [file Image_1.TIF]

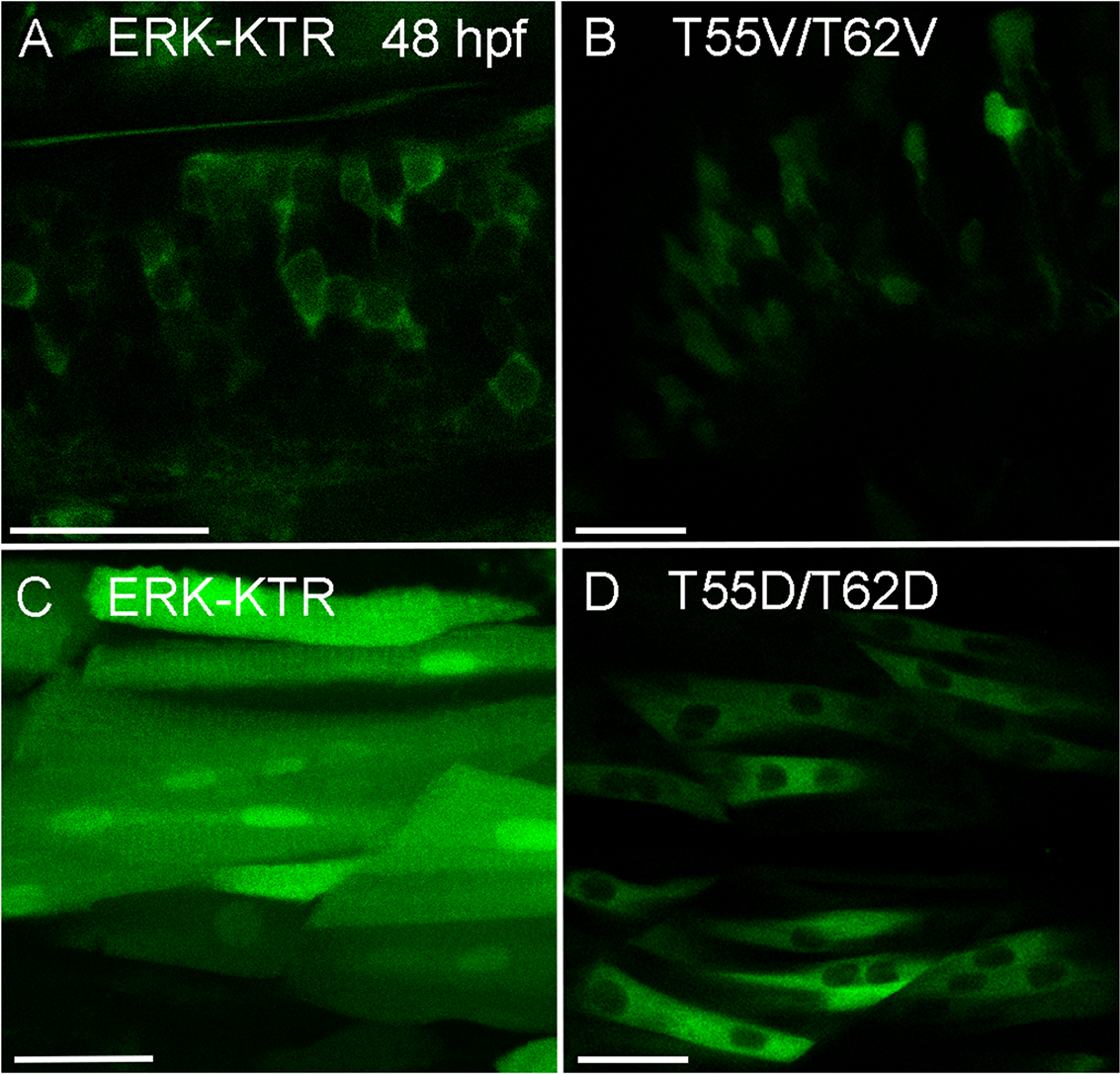

Supplement: Figure S2 — Localization of ERK-KTR control constructs T55V/T62V and T55D/T62D. 48 hpf zebrafish embryos expressing either ERK-KTR-mClover or T55V/T62V and T55D/T62D control reporter constructs. (A) Neural cells within the CNS expressing ERK-KTR-mClover. The reporter localizes to the cytoplasm. (B) The T55V/T62V control reporter is also found in the nucleus of neural cells within the CNS. (C) Muscle cells expressing ERK-KTR-mClover. (D) The phosphomimetic T55D/T62D reporter construct localizes predominantly to the cytoplasm when expressed in muscle cells. All scale bars are 25 μm. Images were recorded on a Leica Sp8 X WLL confocal system and rendered with Adobe Photoshop CS6. [file Image_2.TIF]

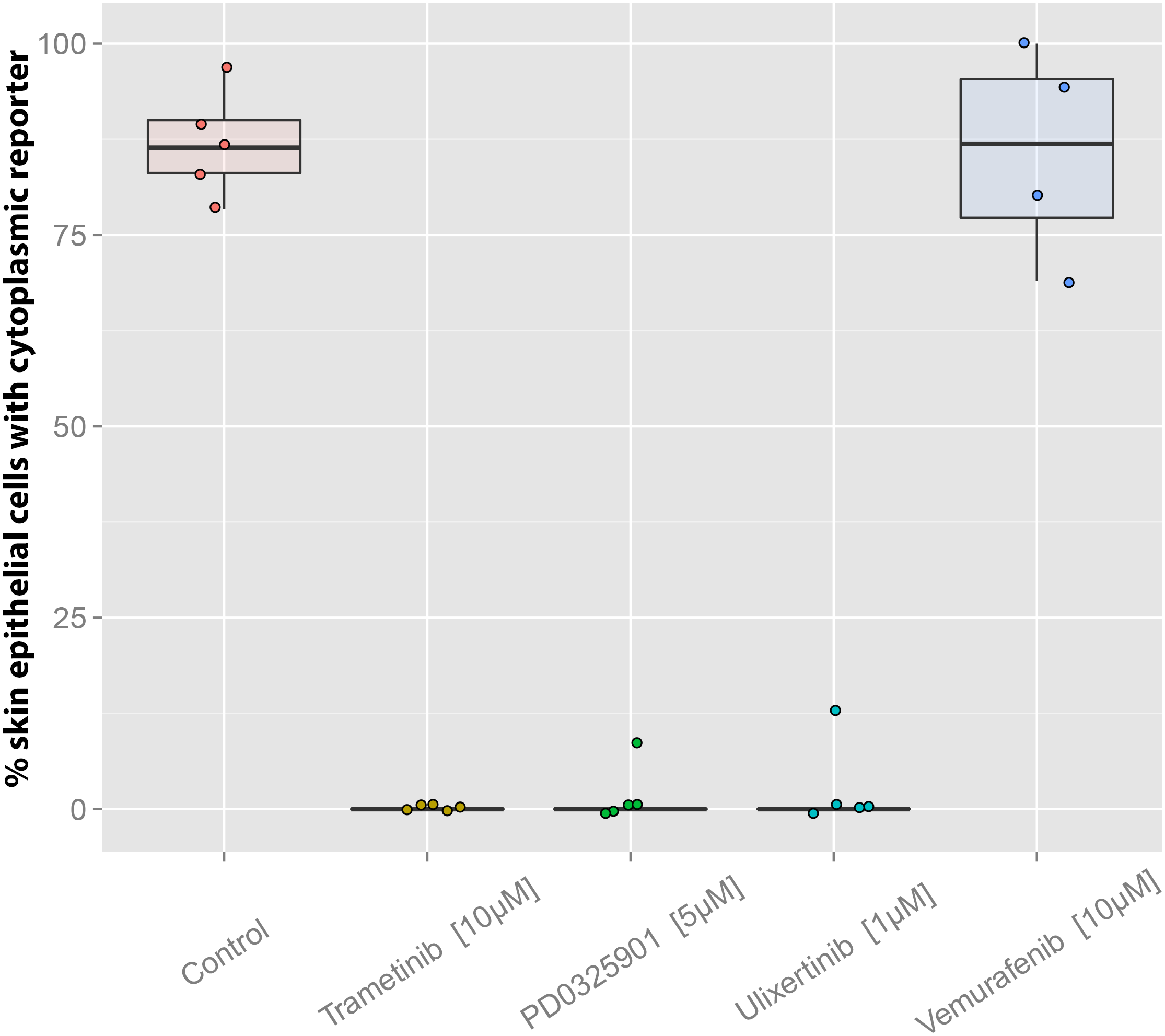

Supplement: Figure S3 — Effects of different inhibitors on Erk activity in DREKA zebrafish. Compounds were applied to F2 DREKA embryos at 29 hpf and quantification was performed on skin epithelial cells at 48 hpf. Percentage of cells with active Erk signaling shown as box plot from left to right: untreated control; ERK1/2 inhibitor: ulixertinib (1 μM); MEK1/2 inhibitor: trametinib (10 μM), PD0325901 (5 μM); B-RAF inhibitor: vemurafenib (10 μM); [each dot represents one embryo; box plot was created using R (r-project.org)]. [file Image_3.TIF]

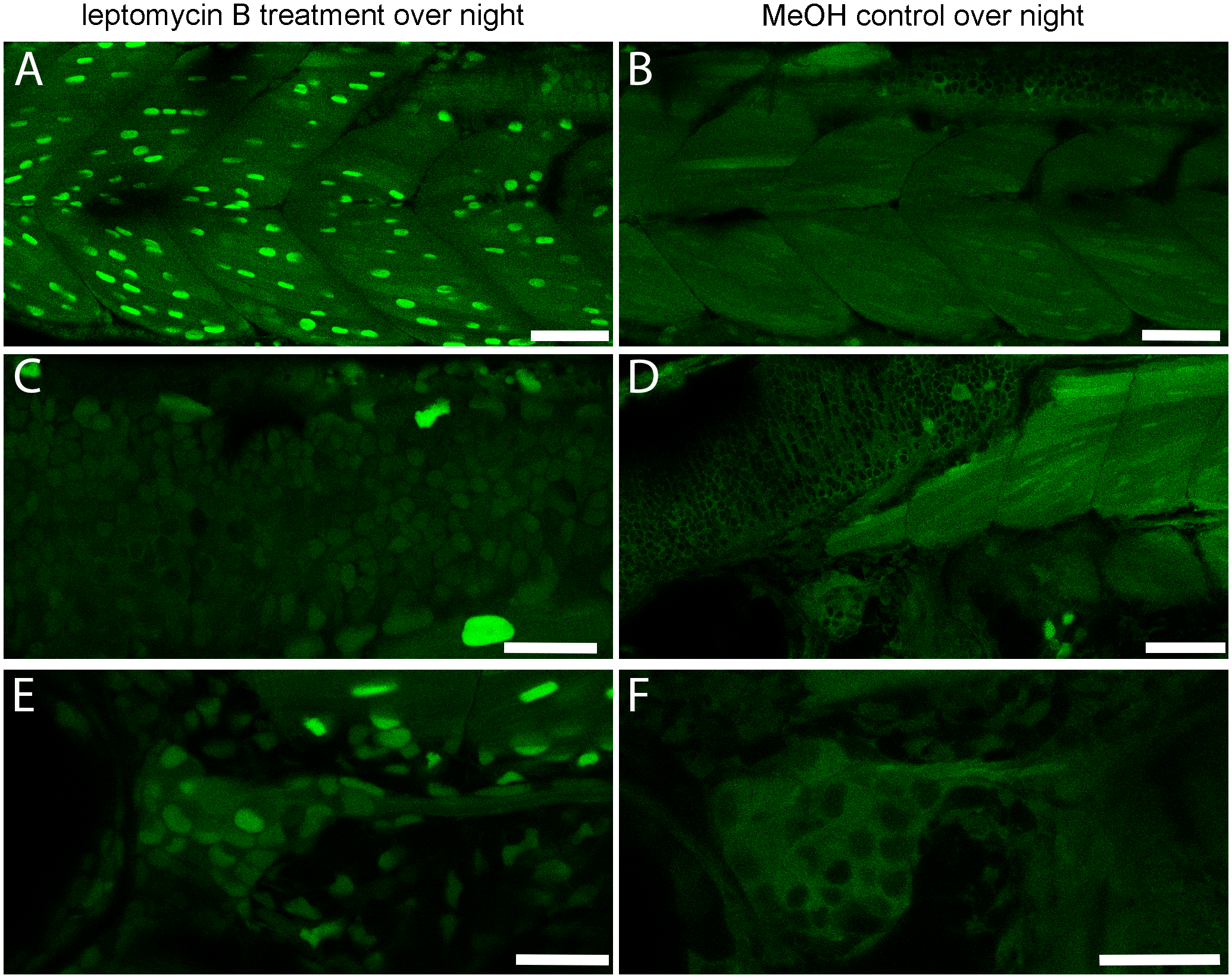

Supplement: Figure S4 — Effects of nuclear export inhibition by leptomycin B. 26 hpf DREKA zebrafish larvae were treated with the nuclear export inhibiting compound leptomycin B at 92 μM for 24 h. (A,C,E) Treated DREKA, (B,D,F) untreated control DREKA. Muscle cells (A,B), neural cells in the hindbrain (C,D), and cranial ganglion cells (E,F) all show increased reporter signal in the nucleus in treated embryos compared to untreated siblings. Scale bars in (A,B,D) are 50 μm, in (C,E,F) 25 μm. Images were recorded on a Leica Sp8 X WLL confocal system using a 25x objective. Images were rendered using Adobe Photoshop CS6. [file Image_4.TIF]
